# A Limited Role for the Cell Cycle Regulator Cyclin A1 in Murine Leukemogenesis

**DOI:** 10.1371/journal.pone.0129147

**Published:** 2015-06-16

**Authors:** Nicole Bäumer, Sebastian Bäumer, Miriam Haak, Steffen Koschmieder, Kai Schönig, Wolfgang E. Berdel, Carsten Müller-Tidow

**Affiliations:** 1 Department of Medicine A, Hematology/Oncology, University of Muenster, Muenster, Germany; 2 Miltenyi Biotec GmbH, Bergisch Gladbach, Germany; 3 Department of Medicine (Hematology, Oncology, Hemostaseology, and SCT), Faculty of Medicine, RWTH Aachen University, Aachen, Germany; 4 Central Institute of Mental Health, Department of Molecular Biology, Heidelberg University, Mannheim, Germany; 5 Dept. of Medicine IV, Hematology and Oncology, University of Halle, Halle, Germany; Emory University, UNITED STATES

## Abstract

The quest for novel therapeutic targets in acute myeloid leukemia (AML) is still ongoing. One of such targets, cyclin A1, was shown to be overexpressed in AML including AML stem cells. However, the function of cyclin A1 in AML is largely unknown, and the data on its impact on patients´ survival remain controversial. Therefore, we developed a transgenic mouse model of stem cell-directed inducible cyclin A1 overexpression and crossed these mice with PML-RARα-knockin mice, which develop an AML M3-like phenotype. To observe the effects of cyclin A1 loss-of-function, we also crossed PML-RARα-knockin mice to cyclin A1-knockout mice. Neither overexpression nor loss of cyclin A1 significantly altered leukemogenesis in PML-RARα-knockin mice. These findings imply that upregulation of cyclin A1 is not essential for leukemogenesis. Our data suggest that cyclin A1 does not represent a suitable target for AML therapy.

## Introduction

Cell cycle regulators are an attractive target for cancer therapy [1]. Cyclin A1 is a catalytic subunit of Cyclin-dependent kinases CDK) 1 and 2 and thus contributes to the regulation of proliferation [2]. It was shown to be expressed in testis and brain [3], and a knock-out of Cyclin A1 in a mouse model lead to infertility of male mice [4] without disturbing other organs. It was therefore thought to mainly function in meiosis.

However, we and others revealed an upregulation of cyclin A1 in AML samples [5–7] as well as in different other cancer entities [8–12]. Although cell cycle proteins including other CDK2-dependent regulators such as cyclin A(2) and cyclin E were expressed in AML [5,6,13], from these candidate genes only high expression of cyclin A1 was correlated with worse overall survival [6]. Elevated levels of cyclin A1 were especially found in samples of AML M3 patients [5,7] that are characterized by the fusion protein PML-RARα. Previously, we found out that cyclin A1 is a direct transcriptional target of PML-RARα function [7]. Moreover, a transgenic mouse model constitutively overexpressing cyclin A1 in myeloid progenitor cells under the control of the human cathepsin G promoter developed a myeloid disease with a low penetrance and long latency [14]. This indicated that cyclin A1 can contribute to the induction of a leukemic phenotype but that at least other cooperative events were necessary to induce a cyclin A1-triggered leukemia. The prominent upregulation of cyclin A1 in PML-RARα-positive AML (this paper and [5]) prompted us to investigate the function of cyclin A1 in AML M3. We took advantage of a previously established AML M3-mouse model that expresses PML-RARα in the cathepsin G gene locus as a knock-in allele and develops an AML-like phenotype with a very high penetrance [15]. In addition, we developed a new transgenic mouse model, in which the expression of cyclin A1 can be induced by tetracycline. We asked the question if the overexpression of cyclin A1 enhances leukemogenesis and whether cyclin A1 expression was necessary for AML.

## Materials and Methods

### Expression analyses

The study was reviewed and approved by the ethics committee of the physician´s chamber of Westfalen-Lippe and the medical faculty of the University of Muenster (2007-524-f-S and 2007-390-f-S) before the study began. AML samples were obtained from the bone marrow of patients with acute myeloid leukemia (AML) at the time of initial diagnosis. The median blast count was 80%. Informed written consent was obtained from all patients.

Reverse transcription and real-time quantitative RT-PCR were performed as described for human cyclin A1 [16,17]. Published microarray data from human bone marrow and blood cells were analyzed using the Leukemia Gene Atlas at http://www.leukemia-gene-atlas.org [18,19]. Cells used for microarray analysis were collected from the purified fraction of mononuclear cells after Ficoll density centrifugation [19].

RNA isolation from sorted murine cells was performed using RNeasy Micro Kit (Qiagen, Hilden, Germany) according to the manufacturer’s protocol.

### Establishment and analysis of cyclin A1-transgenic mice

Using the bacterial artificial chromosome BAC.E11.lacZ and its corresponding plasmid pE11.F3.M.F, the cDNA of human cyclin A1 and luciferase as reporter gene were cloned together with Pbi-1 representing a bidirectional tet-responsive promoter element into the pE11 [20]. This vector also contains two homologous regions corresponding with two regions on the BAC.E11.lacZ flanking a lacZ-gene [20–22]. The construct was recombined into the BAC.E11.lacZ and confirmed with blue-white-selection, PCR, sequencing and Southern Blot (data not shown). One clone could be identified with the complete cDNA of human cyclin A1 and luciferase. This DNA was purified with pulse-field gel electrophoresis and injected into the pronucleus of fertilized zygotes of C57BL6/N mice. The founder lines were bred with the driver mouse line SCL-tTA, which expresses the tetracycline-controlled Transcriptional Activator (tTA) under the control of the Stem Cell Leukemia (SCL) 3’-enhancer [23,24].

In the tTA-system, the expression of human cyclin A1 can be switched off by administration of tetracycline-hydrochloride (Sigma) as described [23]. Luciferase assays were performed following the manufacturer’s protocol (Promega). Retroviral transduction using an rtTA-containing vector was performed as described [25]. For Western blot analysis, peritoneal mast cells were isolated by flushing the peritoneal cavity as described [26] from induced transgenic and control mice. Cyclin A1 Western blots were performed as described [2].

All animal experiments in this study were carried out in strict accordance with the recommendations of the institutional animal care and use committee “Landesamt fuer Natur, Umwelt und Verbraucherschutz NRW”. This study was approved by the Institutional Animal Care and Use Committee and of the local veterinary administration of Muenster (G15/2005, 8.87–51.04.20.09.322, and 87–51.04.2011.A005).

Mice were kept in individually ventriculated (IVC-) Typ II cages (Tecniplast GmbH, Germany) in groups of five mice, in a 12-hour light/dark cycle, with room temperature at 22±2 °C and a relative air humidity of 45–65% (see S1 File: NC3Rs ARRIVE Guidelines Checklist). All mice were allowed free access to water and a maintenance sterile diet. All reasonable efforts were made to ameliorate suffering, including anesthesia using isoflurane inhalation for retro-orbital puncture and isolation of affected mice. Mice were monitored daily for signs of pain or distress. Moribund mice were humanely sacrificed as described below. Study design and biometric planning of each experiment was performed in accordance with a biostatistician. Mice were sacrificed for sample preparation by deep anesthesia via isoflurane inhalation followed by cervical dislocation. For each experiment, the single animal was an experimental unit.

### Breeding

SCL-tTA-tg/cyclin A1-tg mice (FVB/NxC57BL6/N background) or cyclin A1^+/-^ mice (Balb/c background; generously provided by Mark Carrington [2]), respectively, were bred with PML-RARα-knockin-mice (C57BL6/N background; generously provided by Timothy Ley; [15]). Because of the different genetic backgrounds, we compared two groups of littermates of each breeding strategy. SCL-tTA-tg/cyclin A1-tg mice breedings were kept under tetracycline-repression. Cyclin A1 and luciferase expression was induced by withdrawal of tetracycline from the drinking water.

### Analysis of survival

Mice were stated as moribund when they showed certain signs of sickness, i.e. shivering, weight loss, neglected grooming. Moribund animals were euthanized as described above. The results of the survival experiments were analyzed with the log-rank non-parametric and represented as Kaplan-Meier survival curves.

### Flow cytometry and hematological analysis

Preparation of blood, bone marrow or spleen cells for staining and FACS analysis was performed as described previously [25,27]. WBC and blood parameters were analyzed by using the Hemavet multispecies hematology analyzer (Drew Scientific, UK) following the manufacturer´s instruction. Peripheral blood smears were stained according to the May-Grünwald standard procedure. Colony formation assays from total primary bone marrow were carried out essentially as described [25].

## Results and Discussion

**Fig 1:**
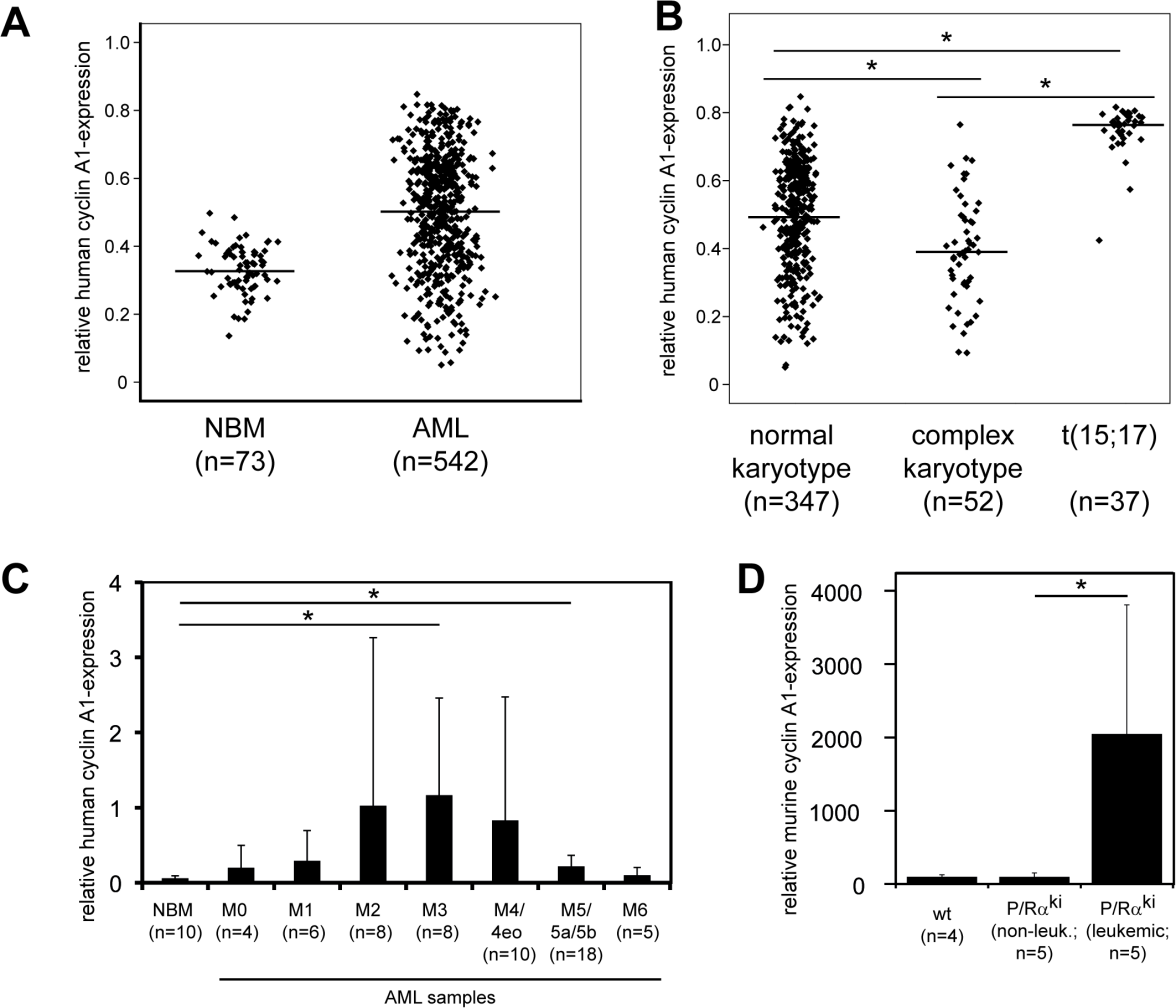
Cyclin A1 expression in human and murine leukemic blasts. A. and B. Cyclin A1 was analyzed in mRNA microarray expression data from the purified fraction of human mononuclear bone marrow cells after Ficoll density centrifugation [18,19]. A. The expression of cyclin A1 was significantly increased in AML blasts compared to normal bone marrow (NBM) (p<0.001, t-test). Shown here are log arbitrary units. B. The expression of cyclin A1 was significantly decreased in AML blasts with complex karyotype and increased in AML M3 blasts compared with normal karyotype and (*p<0.001, t-test). C. Cyclin A1 expression was significantly induced in bone marrow cells from human AML patients with FAB subtype M3 (p = 0.015, t-test) and with FAB subtype M5/5a/5b (p = 0.05, two-tailed t-test) compared to normal bone marrow cells (NBM). Cyclin A1 expression was determined by qRT-PCR and normalized to GAPDH expression level. D. In bone marrow cells of PML-RARα-knockin mice, cyclin A1 expression was significantly upregulated upon a full-blown leukemic phenotype compared to non-leukemic PML-RARα-knockin mice (p = 0.04, t-test).

We and others identified specific upregulation of cyclin A1 in human AML samples (Fig 1A, 1B and 1C), which suggested a function of cyclin A1 in leukemogenesis. This prompted us to analyze cyclin A1 expression in murine AML samples. Indeed, we observed striking and significant upregulation of cyclin A1 expression only in fully developed AML-samples in PML-RARα-knockin mice (Fig 1D) whereas non-leukemic or pre-leukemic mice did not show increased levels of cyclin A1 in the bone marrow (Fig 1D). This indicated that PML-RARα-knockin mice were a suitable model for the analysis of cyclin A1 upregulation in AML.

**Fig 2:**
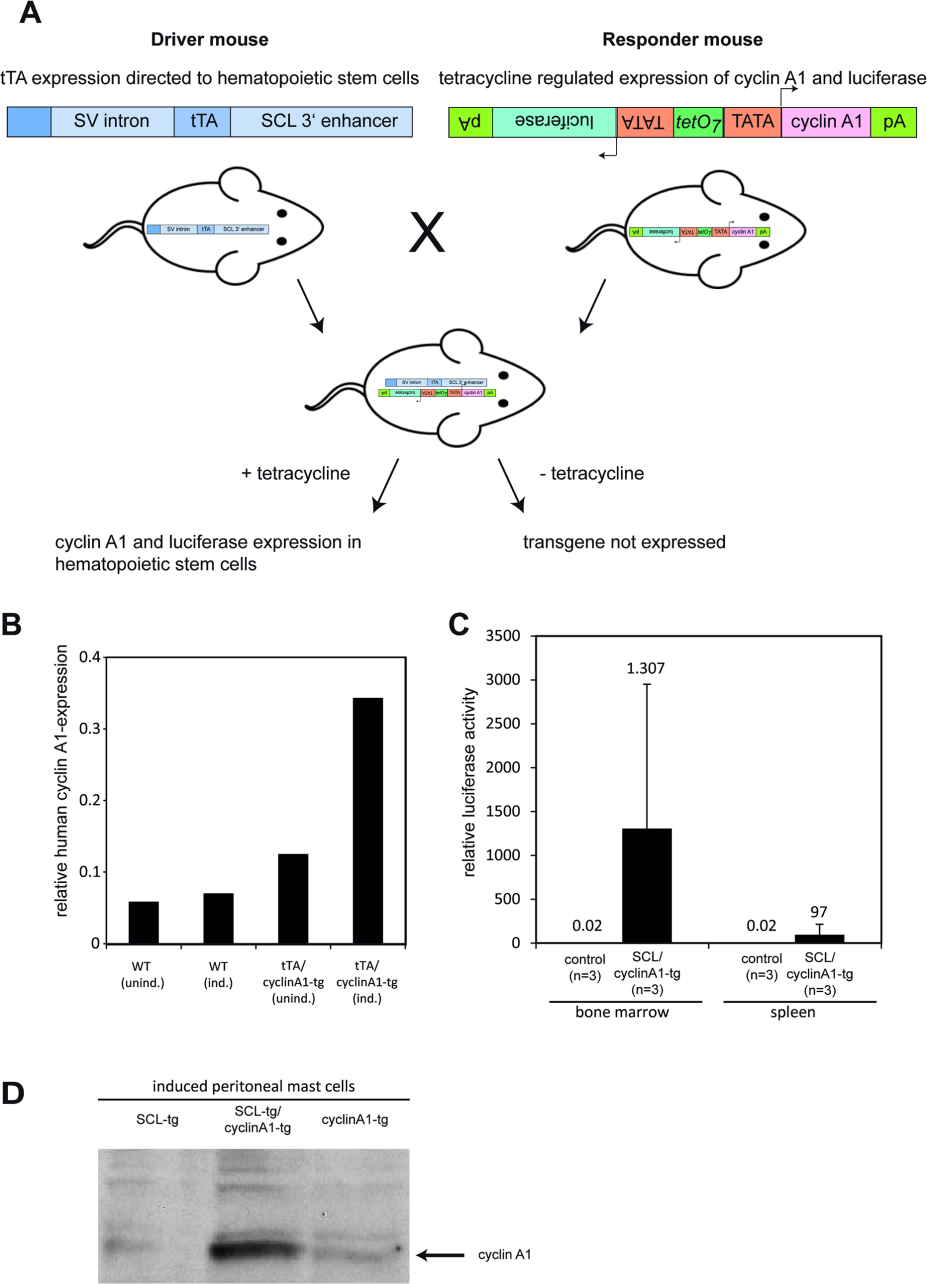
Development of a cyclin A1-transgenic mouse model. A. Schematic overview about the constructs used to develop transgenic mouse lines. The driver mouse line SCL-tTA expresses the tetracycline-dependent transactivator protein (tTA) in hematopoietic stem cells under the control of the stem cell leukemia-factor enhancer [23,24]. In the novel cyclin A1-bi-luciferase responder mouse line, the cDNA of cyclin A1 and luciferase as reporter gene were expressed in absence of tetracycline in parallel and inducibly under control of the bidirectional tTA-responsive promoter element Pbi-1. B. Bars indicate expression levels of human cyclin A1 expression as detected by qRT-PCR in bone marrow cells that were transduced with a retroviral tTA-containing construct and cultured with or without tetracycline in methylcellulose for 10 days (n = 2 for each sample). C. Three months old mice carrying either cyclin A1 alone (control) or together with the driver construct SCL-tTA (SCL-tTAxcyclinA1-tg; n = 3 for each genotype) were induced for seven weeks and investigated for luciferase activity and cyclin A1 mRNA in the bone marrow and spleen. High luciferase activity was only detectable in induced SCL-tTAxcyclinA1-tg bone marrow and spleen cells. Numbers indicate mean luciferase levels.

These data led to the question if ectopic cyclin A1 expression in early hematopoietic stem cells accelerates and/or enhances leukemogenesis. Therefore, we developed an inducible cyclin A1-expressing transgenic mouse model (Fig 2A, right-hand side) that expresses cyclin A1 (Fig 2B) as well as luciferase (Fig 2C) under the control of a bidirectional tetracycline-inducible promoter. We crossed these mice with SCL-tTA mice, a driver mouse line that expressed the tetracyclin-dependent transactivator in early hematopoietic stem and progenitor cells [23,24] and induced expression of cyclin A1 (Fig 2B) by withdrawal of tetracycline. Despite this overexpression, none of double transgenic SCL-tTA/cyclin A1 mice developed a hematopoietic disease during their life time (n = 26, data not shown). Furthermore, hematopoietic stem cell function was not altered, since colony formation of hematopoietic progenitor cells and the number of hematopoietic progenitor and stem cells were not changed (data not shown). This suggests that the AML-like phenotype observed in another transgenic mouse model depended on time, place and the very high level of cyclin A1 expression [14] rather than representing a specific phenomenon. It has long been known that ectopic high level expression of different oncogenes leads to phenotypes different from endogenous expression: For instance, the penetrance of PML-RARα-driven leukemia is dramatically lower when expressed as a transgene [28] compared to expression in the endogenous gene as a knock-in [15]. Moreover, the second known A-type cyclin, cyclin A, which is referred to as cyclin A2 in mice, is highly expressed in bone marrow cells [29]. Conditional knockout of cyclin A2 expression in the bone marrow leads to ablation of the hematopoietic compartment, while the additional absence of cyclin A1 in double-knockout bone marrow did not enhance this phenotype [29]. These results suggest that cyclin A2 has very prominent functions in the bone marrow that the overexpression of cyclin A1 like in our model might not outperform.

**Fig 3:**
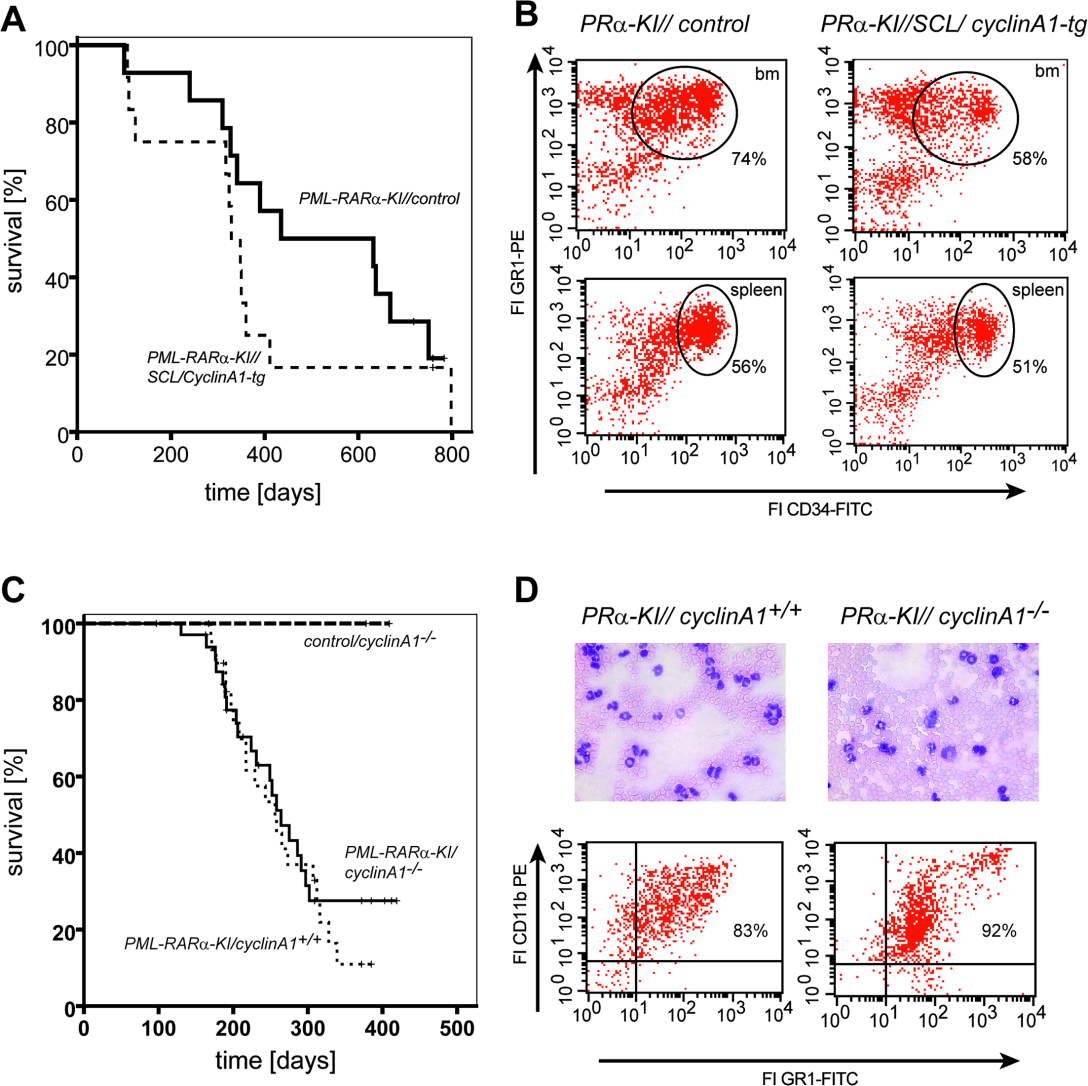
Cyclin A1 expression does not significantly influence PML-RARα-driven leukemogenesis. A. Kaplan-Meier survival curves of heterozygous PML-RARα-knockin mice with (SCL-cyclinA1-tg; n = 12) or without (control; n = 14) ectopic human cyclin A1 expression in the bone marrow. Although there was a trend towards an accelerated disease in the presence of ectopic cyclin A1 expression, latency and penetrance did not differ significantly between the two genotypes (p = 0.282, log-rank test). Also, the phenotype of acute myeloid leukemia was not changed by the presence of cyclin A1 (B). Shown here are examples of FACS analysis of bone marrow (upper panels) and spleen cells (lower panels) of diseased mice. Murine PML-RARα-driven leukemic blasts are characterized by the surface expression of CD34 and GR-1 which does not occur in non-leukemic mice. The number of CD34^+^/GR-1^+^ cells in diseased mice did not alter significantly upon cyclin A1 expression. SCL, Stem Cell Leukemia enhancer driving tTA expression [23]; FI, fluorescent intensity; P/Rα-KI, PML-RARα-knockin mice. C. Kaplan-Meier survival curves of PML-RARα-knockin mice with wild type (PML-RARα-KI/cyclin A1^+/+^; n = 30) or cyclin A1-knockout (PML-RARα-KI/cyclin A1^-/-^; n = 35). Cyclin A1^-/-^ mice without PML-RARα-expression did not develop a lethal phenotype (control/cyclinA1^-/-^; n = 3). Absence of murine cyclin A1 did not affect PML-RARα-driven leukemia. D. The PML-RARα-leukemic phenotype was not altered by the absence or presence of murine cyclin A1. May-Grünwald staining of blood smears showed the same distribution of leukemic blasts and high numbers of differentiated myeloid cells (upper panels). FACS analysis revealed comparable numbers of myeloid cells in the blood (CD11b^+^/GR-1^+^, lower panels).

We bred these SCL-tTA/cyclin A1 double transgenic mice to PML-RARα mice and followed leukemogenesis after induction of cyclin A1 by tetracycline withdrawal. Intriguingly, no significant change in survival (Fig 3A) or leukemia phenotype (Fig 3B) was observed, since the percentage of CD34^+^/GR-1^+^ leukemic blast cells was unchanged in transgenic vs control mice bone marrow (tg: 66+/-18% compared to control: 50+/-21%; t-test: p = n.s.; n = 4 for each genotype) or spleen (tg: 61+/-21% compared to control: 57+/-29%; t-test: p = n.s.; n = 4 for each genotype). This finding suggested a limited function of cyclin A1 upregulation in the initiation as well as in the acceleration of leukemia. The elevated expression of cyclin A1 might rather result from its upregulation by PML-RARα [7] or other AML-driving oncogenes without itself being causative for leukemogenesis.

Although the ectopic expression of cyclin A1 in early hematopoietic cells might not accelerate leukemogenesis, the inhibition or absence of cyclin A1 might preclude disease progression, since higher levels of cyclin A1 were correlated with worse overall survival in AML patients [6]. Therefore, we crossed the PML-RARα-Knock-in mouse line with cyclin A1-knockout mice. Strikingly, mice died by development of AML in presence or absence of cyclin A1, and there was no difference in phenotype or latency of the disease (Fig 3C and 3D). Again, the expression of the other A-type cyclin, cyclin A(2) might compensate for the absence of cyclin A1. Cyclin A(2) has a prominent function in normal hematopoiesis [29] and is also expressed in AML samples [5,6]. Yet the role of cyclin A(2) in AML remains to be determined, but it is tempting to speculate that cyclin A1 and cyclin A2 share functions in leukemic cells. The absence of cyclin A2 lead to decreased liver tumor formation in mice [30]. Therefore, it will be very interesting to investigate leukemogenesis in absence of both A-type cyclins.

In summary, cyclin A1 did not influence the development of murine PML-RARα-driven AML, neither by overexpression in an endogenous setting nor by absence in a complete knock-out model. These results can contribute to the survey for potential leukemic target genes as it seems unlikely that cyclin A1 is an important therapy target in leukemia. On the other hand, cyclin A1 was recently used as a new immunogenic targetable antigen [31]. Using cyclin A1 as a tag to find leukemic cells and to target them by cytotoxic activity of cyclin A1-specific T-cell clones might be a more promising idea than to inhibit the function of cyclin A1.

## Supporting Information

S1 File NC3Rs ARRIVE Guidelines Checklist. (PDF)
